# RanBP1 Couples Nuclear Export and Golgi Regulation through LKB1 to Promote Cortical Neuron Polarity

**DOI:** 10.1016/j.celrep.2018.07.107

**Published:** 2018-09-04

**Authors:** Chiara Mencarelli, Justyna Nitarska, Tim Kroecher, Francesco Ferraro, Katherine Massey, Antonella Riccio, Franck Pichaud

**Affiliations:** 1 Medical Research Council Laboratory for Molecular Cell Biology, University College London, Gower Street, London WC1E 6BT, UK

**Keywords:** neuron polarity, Ran, RanBP1, LKB1, STK25, nuclear export, axon, axonogenesis

## Abstract

Neuronal polarity in the developing cortex begins during the early stages of neural progenitor migration toward the cortical plate and culminates with the specification of the axon and dendrites. Here, we demonstrate that the Ran-dependent nucleocytoplasmic transport machinery is essential for the establishment of cortical neuron polarity. We found that Ran-binding protein 1 (RanBP1) regulates axon specification and dendritic arborization in cultured neurons *in vitro* and radial neural migration *in vivo*. During axonogenesis, RanBP1 regulates the cytoplasmic levels of the polarity protein LKB1/Par4, and this is dependent on the nuclear export machinery. Our results show that downstream of RanBP1, LKB1 function is mediated by the STK25-GM130 pathway, which promotes axonogenesis through Golgi regulation. Our results indicate that the nucleocytoplasmic transport machinery is a main regulator of neuron polarity, including radial migration, and that the regulated export of LKB1 through RanBP1 is a limiting step of axonogenesis.

## Highlights

• The nucleocytoplasmic transport machinery regulates neuron polarity
• Ran/RanBP1 regulates LKB1 availability during axonogenesis
• Ran/RanBP1 couples nucleocytoplasmic transport and Golgi regulation

## Graphical Abstract

**Figure:**
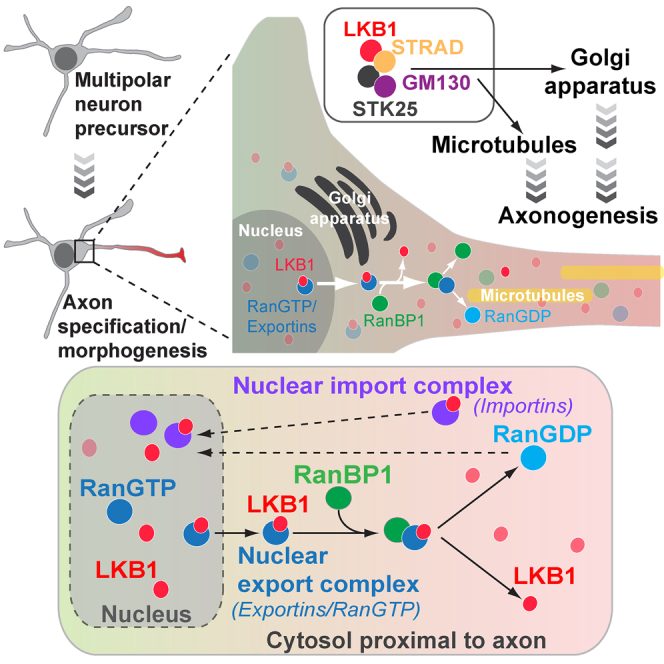

## Teaser

How neurons acquire their polarity is not fully understood. Mencarelli et al. show that the nucleocytoplasmic transport machinery is a key regulator of neuron polarity, including radial migration and axonogenesis. They envisage that regulated nucleocytoplasmic transport of polarity factors may act as a general mechanism of cell polarity in eukaryotes.

## Introduction

Neuron polarity underpins neural circuit formation and function. During cortex development, neuron polarity arises as the neuroepithelial progenitors exit the cell cycle and migrate radially from the ventricular zone toward the cortical plate. Radial migration is initiated by the polarized distribution of N-cadherin, which concentrates at one pole of newly differentiating neurons and defines the position of the first of two opposite neurites (Calderon de Anda et al., 2008, Gärtner et al., 2012, Hatanaka and Murakami, 2002, Pollarolo et al., 2011). This early polarity event influences the latter specification of the axon and dendrites (Noctor et al., 2004). Following radial migration, axonogenesis begins as the cells reach the subventricular zone (SVZ), and neurons acquire their mature morphology as they reach the cortical plate (Hatanaka and Murakami, 2002, Noctor et al., 2004, Shoukimas and Hinds, 1978).

Axon specification is a hallmark of neuron polarity, and in cultured neurons it is regulated by a set of conserved polarity proteins, which includes the partitioning-defective protein Par4/LKB1 (Barnes et al., 2007, Shelly et al., 2007). In cultured neurons, this serine/threonine kinase is necessary to promote polarity through the adaptor kinase STK25, the Golgi protein GM130, and the SAD1/2 kinases, which regulate microtubule stability (Barnes et al., 2007, Matsuki et al., 2010, Shelly et al., 2007). In mammalian cells, a fraction of LKB1 is in the nucleus, and its nuclear export is regulated by the Ran pathway via CRM1/exportin 7 and the exportin co-receptor STRADα (Baas et al., 2003, Dorfman and Macara, 2008). However, whether regulated nuclear export of LKB1 is a regulatory step of neuron polarity has not been examined.

In eukaryotes, nucleocytoplasmic transport of cargo macromolecules through nucleopores depends on a concentration gradient of Ran-guanosine triphosphate (GTP), with high levels of RanGTP in the nucleus, where the Ran-guanine nucleotide exchange factor (GEF) RCC1 is localized (Bischoff and Ponstingl, 1991, Nemergut et al., 2001), and low levels in the cytosol, where Ran-guanosine diphosphate (GDP) is the predominant form. In the cytosol, the karyopherin proteins importin-α and importin-β1 form a cargo receptor, which drives cargo import to the nucleus. In the nucleus, RanGTP promotes cargo release by binding to the importins (Bischoff and Görlich, 1997). Conversely, nuclear export of cargoes relies on the assembly of export complexes that include RanGTP, a karyopherin that acts as a cargo receptor, and the cargo. In the cytosol, Ran-binding protein 1 (RanBP1) dissociates the export complex, releasing the cargo and allowing the recycling of RanGTP to RanGDP through Ran-GTPase-activating protein (GAP) (Bischoff and Ponstingl, 1995, Kuhlmann et al., 1997, Lounsbury and Macara, 1997, Seewald et al., 2003). Thus, RanBP1 regulates cargo release during nuclear export and maintains low levels of RanGTP in the cytosol, both of which are essential for nuclear import.

In addition to their function in mediating nucleocytoplasmic transport, Ran and RanBP1 regulate spindle assembly (Kalab et al., 1999, Zhang et al., 2014) and primary cilium initiation (Fan et al., 2011). Furthermore, Ran and RanBP1 regulate nerve repair in peripheral sensory neurons (Yudin et al., 2008). In these neurons, RanBP1 is translated at the site of axonal injury and allows for importin-α and importin-β1 to form a complex that is linked to the microtubule motor dynein. This complex is believed to enable retrograde signaling by transporting signaling cargoes back to the nucleus (Lai et al., 2008, Yudin et al., 2008). Finally, recent work has shown that RanGTP can be detected at the tip of extending neurites, where it regulates acentrosomal microtubule nucleation through TPX2 (Chen et al., 2017). In this context, whether RanBP1-dependent cargo unloading during nucleocytoplasmic transport plays a role in regulating neuron polarity remains unknown.

## Results

### Components of the Ran Pathway Regulate Axonogenesis in *Drosophila*

To identify factors regulating axon growth *in vivo*, we performed a genetic screen in the *Drosophila melanogaster* larval visual system, which is referred to as the Bolwig’s organ. This bilateral sensory organ consists of approximately 12 photoreceptors that project axons to the optic lobe during the early larval stages (Figure S1A). As the larva grows, the Bolwig’s nerve elongates proportionally in a manner that is similar to that of the sciatic nerve in humans after post-embryonic development. A screen of 4,000 RNAi lines covering all cytoskeletal regulators, kinases and phosphatases, the small GTPases and their regulators, as well as transmembrane proteins predicted in the fly genome, revealed 83 candidate genes that affected Bolwig’s nerve growth (Table S1). We identified among the genes that led to a shortening of the Bolwig’s nerve several regulators of the Ran pathway, including Ran (Figures S1B–S1I), indicating a role for this pathway during axon growth.

### Localization of Ran, RCC1, RanGAP, and RanBP1 in Mammalian Cortical Neurons

**Figure 1:**
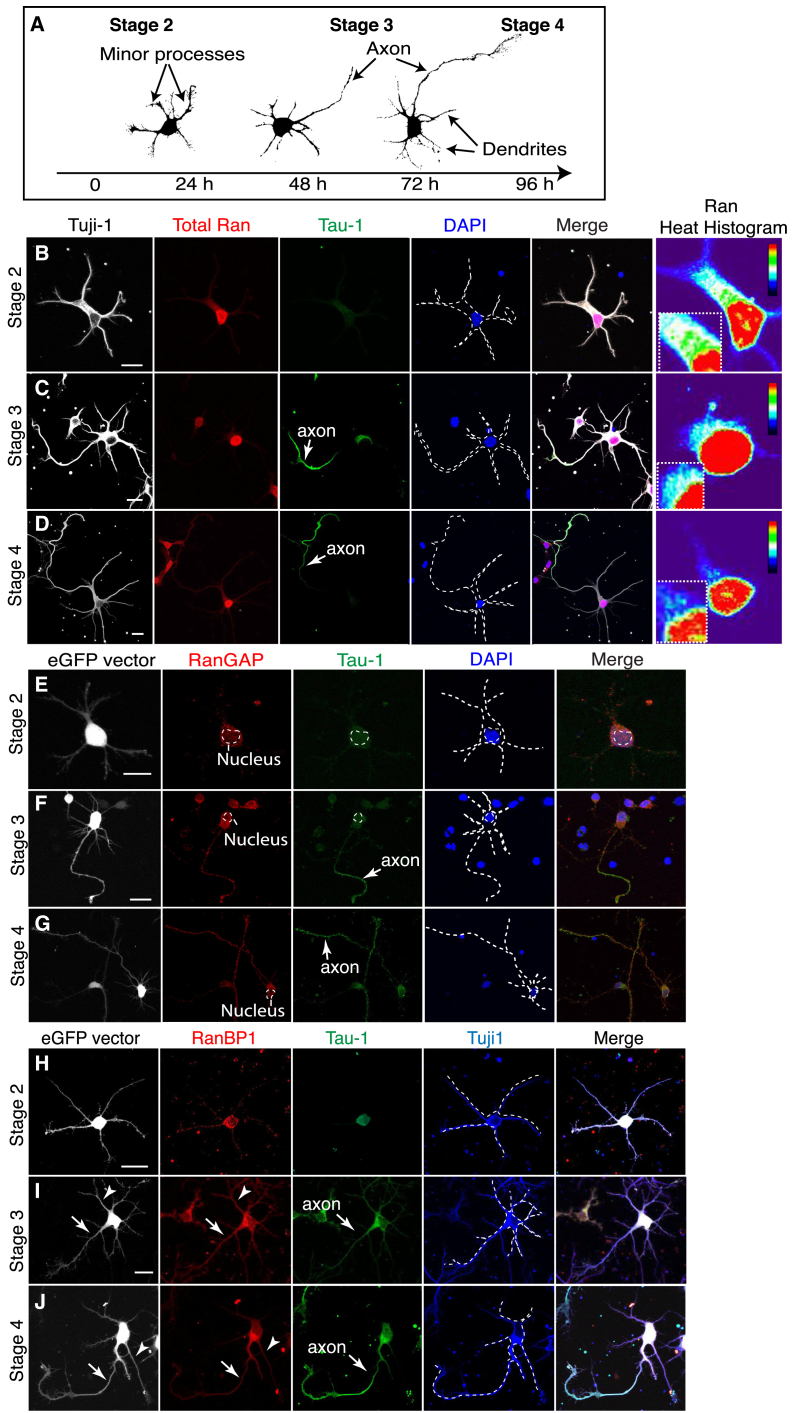
Subcellular Localization of Ran, RanGAP, and RanBP1 in Cortical Neurons (A) Successive stages of rat cortical neuron polarization *in vitro*. Neurons transfected with the control *eGFP*-reporter plasmid were imaged at different stages of development, and z stacks of individual cells were skeletonized using ImageJ (NIH). (B–D) Rat cortical neurons stained for Tuji-1, Ran, and Tau-1 at Stage 2 (B), Stage 3 (C), and Stage 4 (D). The right column shows a heat histogram of Ran staining intensity. Scale bar, 10 μm. (E–G) Neurons stained for eGFP, RanGAP, Tau-1, and DAPI at Stage 2 (E), Stage 3 (F), and Stage 4 (G). Scale bar, 10 μm. (H–J) Neurons stained for eGFP, Tuji-1, Tau-1, and RanBP1 at Stage 2 (H), Stage 3 (I), and Stage 4 (J) using a previously validated RanBP1 antibody (Yudin et al., 2008). A white arrow points to RanBP1 staining in the axon and the arrowhead points to RanBP1 staining in a dendritic process.

Next, we investigated whether regulated Ran-dependent nucleocytoplasmic transport promotes axon growth in vertebrate neurons using neonatal (postnatal day 0 [P0]) rat cortical neurons. Cortical neurons differentiate *in vitro* following four defined morphological stages (Figure 1A). During the first 12 to 24 hr, cells extend short processes (stages 1 and 2). After 36 hr, one process extends rapidly and acquires axon-specific markers (stage 3). This is followed by further axon elongation and dendrite development between 72 and 96 hr (stage 4) in culture (Dotti et al., 1988).

We studied the expression pattern of the Ran pathway proteins during neuron polarization. In polarized neurons (stages 3 and 4), Ran was enriched in the nucleus and detected at lower levels in the cell body, dendrites, and the axon (Figures 1B–1D). RanGAP was distributed evenly throughout the cytoplasm, dendrites, and the axon (Figures 1E–1G). Using an antibody previously validated in peripheral sensory neurons (Yudin et al., 2008), we found that RanBP1 was localized in the cytosol, axon, and dendrites (Figures 1H–1J). However, we also found that a fraction of RanBP1 associated with the trans-Golgi network (TGN) using two independent antibodies. The specificity of these two antibodies was confirmed in cortical neurons using small interfering RNA (siRNA) (Figures S2A–S2F). Consistent with RanBP1 being associated with the TGN, scattering of the Golgi apparatus using brefeldin A (BFA) led to the scattering of RanBP1 (Figures S2G and S2H). To further investigate the link between the Golgi apparatus and RanBP1, we performed subcellular fractionation of rat brain cortices. A first fractionation based on sedimentation velocity was used to enrich for Golgi markers, followed by equilibrium sedimentation of the pooled peak fractions to further resolve the Golgi-enriched peak. Western blotting using antibodies targeting a number of Golgi proteins showed that a fraction of RanBP1 co-fractionates with the Golgi compartments (Figure S2I). Our results show that RanBP1 is enriched in the cytosol, including the Golgi apparatus, proximal to the nascent axon.

### Nucleocytoplasmic Transport Regulates Axon Specification

**Figure 2:**
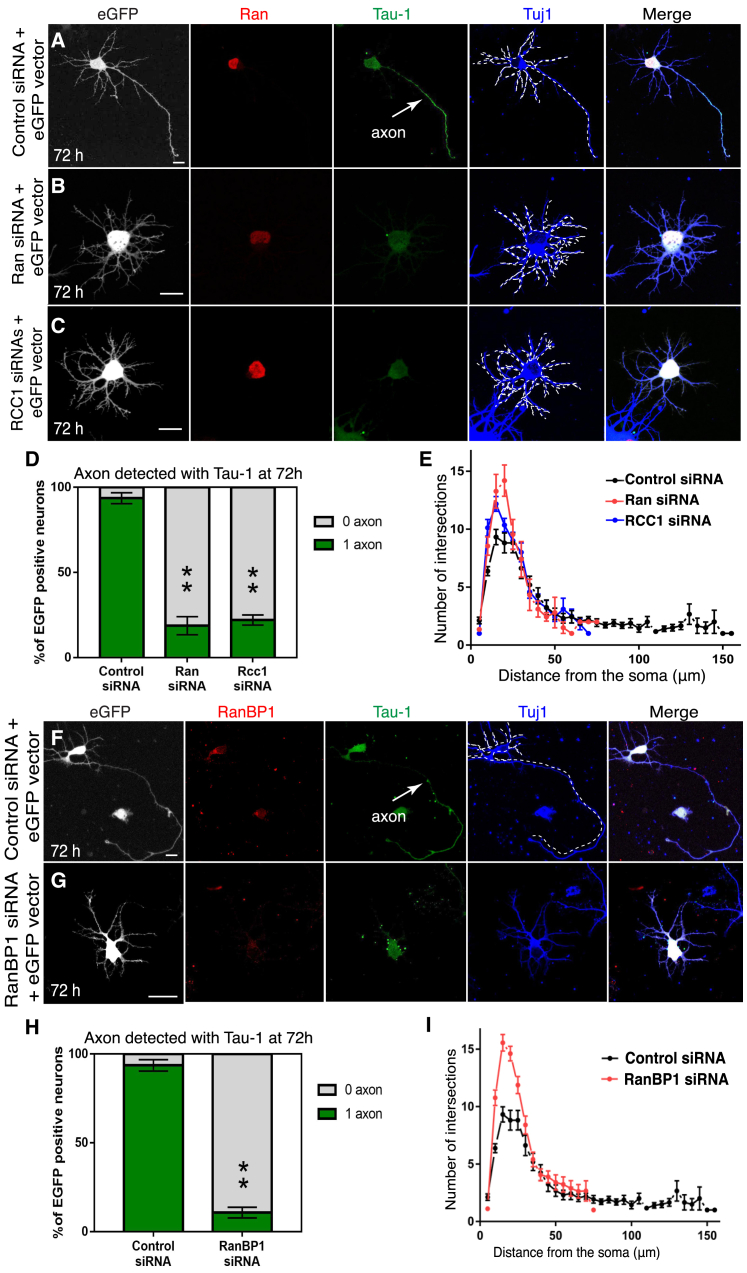
The Ran Pathway Is Required for Rat Cortical Neuron Polarization (A) 72 hr cortical neuron co-transfected with a control siRNA and an *eGFP* reporter plasmid. (B) 72 hr cortical neuron co-transfected with *ran* siRNAs and an *eGFP*-reporter plasmid (scale bar, 10 μm). (C) 72 hr cortical neuron co-transfected with *rcc1* siRNAs and an *eGFP*-reporter plasmid (scale bar, 10 μm). (D) Neuron polarity was scored at 72 hr after transfection using the axonal marker Tau-1. The mean percentage of neurons with 0 or 1 axon over three independent replicates was determined for each condition: control siRNA (n = 31 neurons), *ran* siRNA (n = 32 neurons), and *rcc1* siRNA (n = 28 neurons). After converting percentages to arcsin values, two-tailed unpaired t tests were performed, comparing control siRNA to *ran* siRNA (p = 0.0019) and Rcc1 siRNA (p = 0.0014). (E) Sholl analysis of *ran* siRNAs neurons (n = 32 neurons), *rcc1* siRNAs neurons (n = 28 neurons), and control scrambled siRNA neurons (n = 31 neurons) was performed 72 hr after transfection. A statistically significant increase in intersection numbers was observed for *ran* knockdown between 10 and 20 μm from the cell body (at 10 and 15 μm ^∗^p < 0.05; at 20 μm ^∗∗^p < 0.01) and for *rcc1* knockdown between 10 and 15 μm from the cell body (at 10 and 15 μm ^∗^p < 0.05). No intersections were detected beyond 60 μm from the soma in the *ran* or *rcc1* knockdown neurons, whereas in the wild-type at least one intersection could be readily detected 90 μm from the soma. Error bars represent SEMs. Statistics were calculated by unpaired two-tailed Student’s t test. (F and G) 72 hr cortical neurons co-transfected with (F) control siRNA and an *eGFP*-reporter plasmid or with (G) siRNAs targeting *ranBP1* and an *eGFP*-reporter plasmid and stained for RanBP1, Tau-1, and Tuj1. Ninety percent of the *ranBP1* knockdown neurons failed to polarize by 72 hr (n = 38 neurons analyzed from at least three separate transfection experiments). In contrast, 94% of control neurons are polarized (n = 31 neurons, from at least three separate transfection experiments). (H) The percentage of 72 hr neurons with 0 or 1 axon over three independent replicates was determined for each condition: control siRNA (n = 31 neurons) and *ranBP1* siRNA (n = 38 neurons). After converting percentages to arcsin values, a two-tailed unpaired t test was performed, comparing control siRNA to *ranBP1* siRNA (p = 0.00084). (I) Sholl analysis of *ranBP1* siRNAs neurons (n = 38) and control siRNA neurons (n = 31 neurons) was performed 72 hr after transfection. A statistically significant increase in the number of intersections was observed between 5 and 25 μm from the cell body (between 5 and 20 μm ^∗∗∗^p < 0.001; at 25 μm ^∗^p < 0.05, two-tailed unpaired Student’s t test). No intersections were detected beyond 75 μm from the soma in the *ranBP1* knockdown neurons, whereas in the case of wild-type neurons, at least one intersection could be readily detected 90 μm from the soma. Error bars represent SEMs.

To study whether nucleocytoplasmic transport affected neuron polarity, we inhibited *ran* expression using siRNA. Most of the cortical neurons (∼80%) transfected with *ran* siRNA failed to specify their axon after 72 hr and showed short and hyperbranched neurites, whereas neurons transfected with scrambled siRNA (scrambled siRNA/GFP) differentiated normally (Figures 2A, 2B, 2D, 2E, S3A, and S3B). A similar phenotype was observed when the expression of the RanGEF RCC1 (Figures 2C–2E) and RanBP1 (Figures 2F–2I and S3C–S3E) was reduced.

To complement our analysis of the *ranBP1* phenotype, we examined formation of the axonal initial segment (AIS) using ankyrin-G antibodies. Reducing the expression of RanBP1 using siRNA abolished the AIS in a significant proportion of neurons when compared to control neurons. Only 20% of *ranBP1*-siRNA neurons had one AIS compared to 90% of neurons treated with scrambled siRNA (Figures S4A–S4C). This result indicates that the inhibition of RanBP1 expression largely prevents axon specification and morphogenesis in mammalian cortical neurons. To rule out potential effects on cell viability, RanBP1 levels were maintained low for 15 days using repeated rounds of siRNA transfections. Under these conditions, only 10% of RanBP1-siRNA neurons presented one axon compared to 80% of scrambled RNAi neurons with one axon (Figures S4D–S4F). Taken together, these data indicate that the Ran pathway and RanBP1 regulate axonogenesis in cortical neurons.

Next, to investigate whether Ran, RCC1, and RanBP1 link nucleocytoplasmic transport to the establishment of neuronal polarity, we asked whether hRanBP1[E37K], a mutated form of human-RanBP1 that does not bind to Ran-GTP (Petersen et al., 2000), could rescue the *ranBP1* siRNA-mediated axonogenesis defects. As a control, we used hRanBP1::GFP, which localizes in the cytosol and rescues neuronal polarity in 72% of transfected cells (Figures S5A, S5B, and S5D). In contrast, expression of hRanBP1[E37K]::GFP, which also localizes in the cytosol (Figure S5C), exacerbated the axonless phenotype and failed to rescue axonogenesis in *ranBP1*-deficient neurons (Figure S5D). These experiments confirm that the Ran-dependent nucleocytoplasmic transport pathway regulates polarity in rat cortical neurons. Our results show that in these cells, RanBP1 is a key regulator of axonogenesis and dendritic arborization.

### RanBP1 Regulates the Nuclear Export of Par4/LKB1

**Figure 3:**
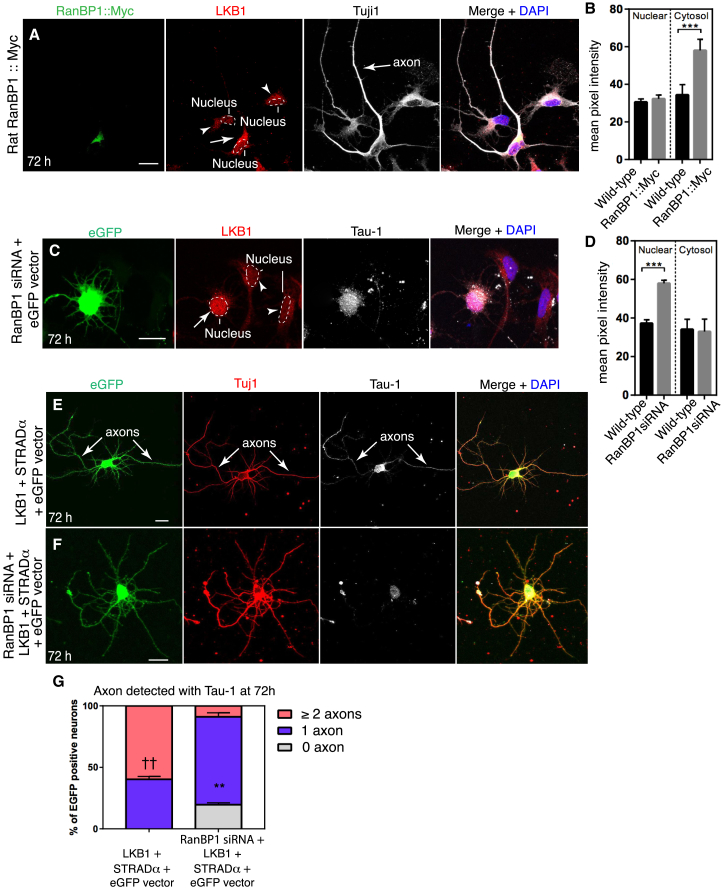
RanBP1 Is Required for LKB1-Dependent Axonogenesis (A) LKB1 localization in a representative 72 hr rat cortical neuron overexpressing RanBP1::Myc, compared to non-transfected neurons (open triangles). Scale bar, 10 μm. (B) Quantification of the LKB1 signal intensity in the nucleus and cytosol of neurons overexpressing RanBP1::Myc (n = 23 neurons), compared to neighboring non-transfected neurons (n = 29 neurons). Wild-type nuclear: 30.55 ± 1.69; RanBP1::Myc nuclear: 32.32 ± 1.94. Error bars represent SEMs (p = 0.50, two-tailed unpaired t test. Wild-type cytosol: 34.37 ± 1.72; RanBP1::Myc cytosol: 58.03 ± 1.70. Error bars represent SEMs (p < 0.0001, two-tailed unpaired t test). (C) LKB1 localization (white arrow) in representative RanBP1 deficient 72 hr neurons compared to non-GFP neurons. Scale bar, 10 μm. (D) Quantification of LKB1 signal intensity in the nucleus and cytosol of neurons 72 hr after siRNA-mediated knock down of *ranBP1* (n = 20) compared to non-GFP neurons (n = 19). Wild-type nuclear: 37.25 ± 1.81; *ranBP1*-siRNA nuclear: 57.98 ± 1.61. Error bars represent SEMs (p = 0.0001, two-tailed unpaired t test). Wild-type cytosol: 34.13 ± 1.35; *ranBP1*-siRNA cytosol: 32.97 ± 1.71. Error bars represent SEMs (p = 0.60, two-tailed unpaired t test). (E) Co-expression of *LKB1* and *STRADα* together with an *eGFP* reporter plasmid in rat cortical neurons. White arrows indicate axons. (F) *ranBP1*-siRNA neurons overexpressing LKB1 and STRADα. (G) The mean percentage of 72 hr neurons with 0, 1, or ≥2 axons over three independent replicates was determined for each condition: overexpression of LKB1+STRADα (n = 32 neurons) and overexpression of LKB1+STRADα with knock down of *ranBP1* (n = 34 neurons). After converting percentages to arcsin values, two-tailed unpaired t tests were performed. Comparing LKB1+STRADα against LKB1+STRADα for the proportion of neurons with 1 axon (p = 0.00187, ^∗∗^) and 2 axons (p = 0.00790, ††).

A potential cargo of the Ran pathway that could mediate RanBP1 function during axonogenesis is LKB1 (Barnes et al., 2007, Shelly et al., 2007). Because we found that RanBP1 is localized proximal to the nascent axon, we hypothesized that it may promote axon specification by unloading LKB1 from the nuclear export complex at this location. To test this hypothesis, we overexpressed RanBP1 in cortical neurons and analyzed the subcellular distribution of LKB1. Expression of RanBP1 led to substantial accumulation of LKB1 in the cytosol, relative to controls (Figures 3A and 3B). Conversely, compared to wild-type neurons, inhibition of RanBP1 levels using siRNA led to decreased cytoplasmic accumulation and increased nuclear levels of LKB1 (Figures 3C and 3D). Thus, we concluded that RanBP1 regulates the distribution of LKB1 between the nucleus and cytosol during neuron polarization.

Overexpression of LKB1 and STRADα induces supernumerary axons in cultured neurons (Figure 3E) (Barnes et al., 2007, Shelly et al., 2007). To further study the relation between LKB1 and RanBP1, we asked whether RanBP1 is required for this phenotype. Further supporting the hypothesis that RanBP1 plays a key role in regulating the availability of LKB1 during neuron polarity, we found that overexpression of LKB1 and STRADα did not generate additional axons when RanBP1 expression was also decreased, and in 20% of the cases, no axon could be detected (Figures 3E–3G). These results show that RanBP1-dependent unloading of LKB1 from the nuclear export complex is a key step in regulating LKB1 function during axonogenesis.

### RanBP1 Regulates Neuron Polarity through the LKB1-STRAD-STK25 Pathway

**Figure 4:**
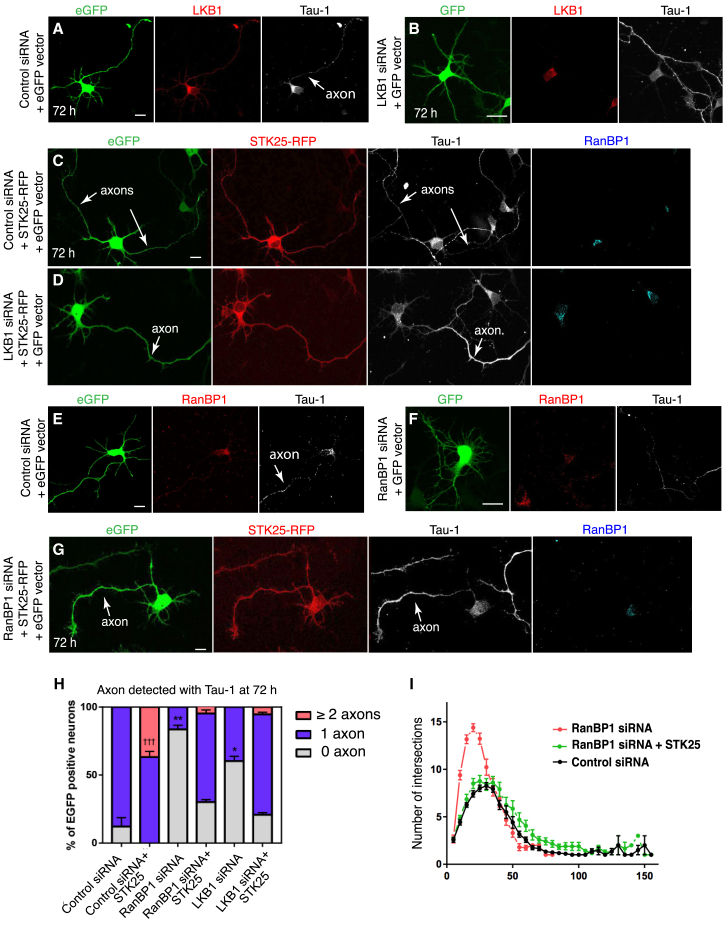
LKB1 Is the Main Cargo Regulated by the Ran/RanBP1 Pathway during Axonogenesis (A and B) Rat cortical neuron transfected with control siRNAs and the *eGFP*-reporter (A) and siRNAs targeting *LKB1* and the *eGFP*-reporter plasmid (B). (C) 72 hr cortical neuron overexpressing STK25-red fluorescent protein (RFP) and scrambled siRNA together with an *eGFP*-reporter plasmid. (D) 72 hr cortical neuron overexpressing STK25-RFP and *ranBP1*-siRNA together with the *eGFP*-reporter plasmid. (E and F) 72 hr cortical neuron transfected with scrambled siRNA and the *eGFP*-reporter (E) and 72 hr cortical neuron transfected with siRNAs targeting *ranBP1* and the *eGFP*-reporter plasmid (F). (G) 72 hr cortical neuron transfected with siRNAs *ranBP1*, *STK25-RFP*, and an *eGFP*-reporter plasmid. (H) The mean percentage of 72 hr neurons with 0, 1, or ≥2 axons over three independent replicates were determined for each condition. The absence of axon in *ranBP1* and *LKB1* siRNA neurons is rescued by expressing *STK25-RFP*. At least 24 neurons were scored for the number of axons for each condition. (I) Sholl analysis of siRNA control (n = 15), *ranBP1*-siRNAs neurons (n = 18), and *ranBP1*-siRNAs neurons rescued by overexpressing *STK25-RFP* (n = 21) was performed 72 hr after transfection. A statistically significant decrease in intersection numbers was observed for the rescue experiment between 10 and 25 μm from the cell body (at 10, 15, 20, and 25 μm ^∗^p < 0.001). No intersections were detected beyond 60 μm from the soma in the *ranBP1* knockdown neurons, whereas in the rescued experiment and control siRNA, intersections reaching 90 μm could be readily detected. Comparing control siRNA and *ranBP1* siRNA*STK25*(+) shows no statistically significant differences in the first 70 μm from the cell soma. Comparing control siRNA and *ranBP1*-siRNA shows statistically significant differences at 10, 15, 20, and 25 μm from the cell (p < 0.0001). Error bars represent SEMs. Statistics were calculated by two-tailed unpaired Student’s t test.

LKB1 function during axon specification is partly mediated by STK25, and overexpression of STK25 in LKB1-deficient cells can restore axon specification (Figures 4A–4D and 4H) (Matsuki et al., 2010). Our work raises the possibility that LKB1 is the main cargo that mediates RanBP1 function during neuronal polarization. Consistent with this hypothesis, overexpression of STK25 in neurons where RanBP1 expression has been decreased ameliorates the polarity phenotype, with 65% of *ranBP1*-siRNA(+)STK25 neurons presenting an axon, compared to 12% of neurons transfected with *ranBP1*-siRNA (Figures 4E–4H). In addition, the hyperbranched dendrite phenotype of RanBP1-siRNA neurons was also suppressed when expressing STK25 (Figure 4I). These findings indicate that during neuron polarization, RanBP1 controls the availability of LKB1 in the cytosol and enables axonogenesis through the STK25 pathway.

### RanBP1 Regulates Radial Migration of Neurons during Embryonic Cortical Development

To investigate the role of the nucleocytoplasmic transport machinery during neuronal differentiation and radial neural migration *in vivo*, we turned to the developing mouse cortex. We found that the Ran, RanGAP, and RanBP1 expression patterns in *vivo* resembled those found in cultured neurons (Figures S6A–S6C). RanBP1 and RanGAP were mostly localized to the cytosol (Figure S6A and S6B), while Ran was enriched in the nucleus relative to the cytosol (Figure S6C).

**Figure 5:**
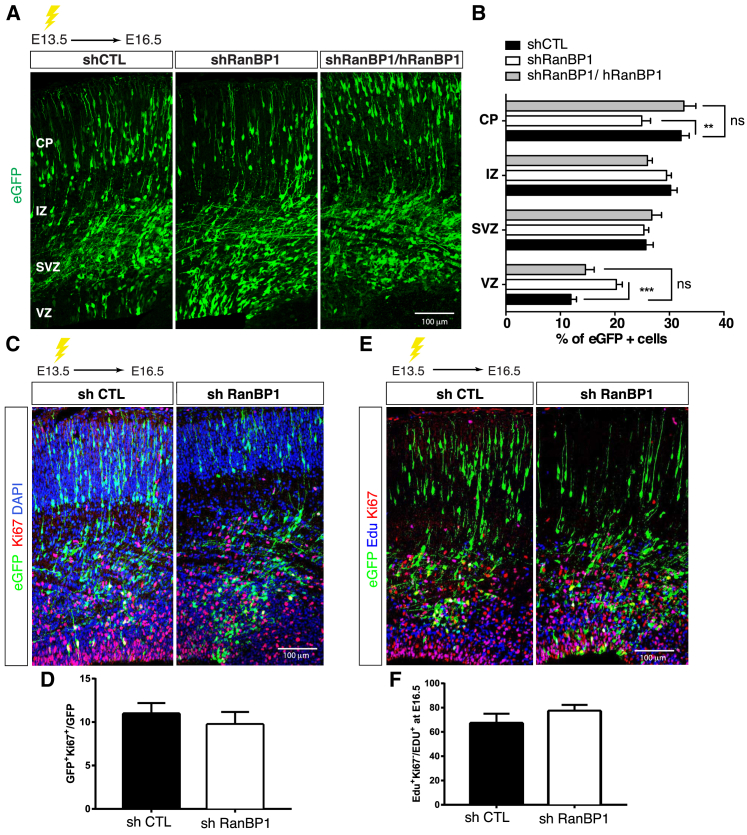
RanBP1 Regulates Neuronal Migration *In Vivo* (A) E13.5 mice were electroporated with shCTL, sh*ranBP1* (sh*ranBP1*_1), or sh*ranBP1* + h*ranBP1* and analyzed at E16.5. Electroporated cells are GFP^+^ (green). (B) Quantification of the distribution of electroporated cells in indicated cortical areas represented as the percentage of GFP^+^ cells. Embryos were obtained from three independent electroporation experiments (shCTL [n = 14], shRanBP1 [n = 17], sh*ranBP1*+h*ranBP1* [n = 10]). Neuronal migration was analyzed using two-way ANOVA with Tukey’s post-hoc test. p values are as follows: for VZ: shCTL versus sh1 0.0003, shCTL versus sh1+Rescue 0.9757, sh1 versus sh1+Rescue 0.1526; for SVZ: shCTL versus sh1 > 0.9999, shCTL versus sh1+Rescue > 0.9999, sh1 versus sh1+Rescue 0.9999; for IZ: shCTL versus sh1 > 0.9999, shCTL versus sh1+Rescue 0.6250, sh1 versus sh1+Rescue 0.8169; for CP: shCTL versus sh1 0.0040, shCTL versus sh1+Rescue > 0.9999, sh1 versus sh1+Rescue 0.0063. CP, cortical plate; IZ, intermediate zone; SVZ, subventricular zone; VZ, ventricular zone. (C) E13.5 embryos were subjected to *in utero* electroporation and analyzed at E16.5. Representative images of coronal mouse embryonic sections stained for GFP and Ki67 antibodies. Scale bar, 100 μm. (D) Quantification of cells expressing Ki67. Data are from 3 independent experiments and 10 embryos per condition. (E) Representative images of coronal mouse embryonic sections stained for GFP, Edu, and Ki67 antibodies. (F) The proliferation index was measured as GFP^+^Ki67^+^/GFP^+^ cells. No significant difference was detected when comparing shCTL (10.96 ± 1.24) to sh*ranBP1* (9.72 ± 1.45). The rate of cell-cycle exit was measured as the ratio of Edu^+^Ki67^−^/Edu^+^ cells. No significant difference was detected when comparing shCTL (67.13 ± 7.91) to sh*ranBP1* (77.14 ± 5.18).

During cortex development, the establishment of neuronal polarity is a prerequisite for radial migration. To test RanBP1 function during cortex development, we generated three independent small hairpin RNA (shRNA) plasmids. All three shRNAs efficiently inhibited RanBP1 levels in cell lines and in the embryonic mouse cortex (Figures S7A–S7C). Mouse brains were electroporated at embryonic day 13.5 (E13.5), and radial migration was analyzed after 72 hr by counting the number of GFP-expressing neurons that had reached the cortical plate. *In utero* electroporation using the two most efficient *ranBP1*-shRNA constructs (shRNA2 and -3) resulted in a strong reduction in GFP^+^ neurons when compared to the control shRNA (small hairpin control [shCTL]), which may be due either to cell death or defects of neural progenitor proliferation. Loss of GFP^+^ cells was not detected using the less efficient *ranBP1*-shRNA construct (*ranBP1*-shRNA1), allowing us to investigate the role of RanBP1 during cortical radial migration. A significant proportion of neurons electroporated with *ranBP1*-shRNA1 accumulated within the ventricular (VZ) and SVZ and failed to reach the cortical plate (CP) (Figures 5A and 5B). This was not due to off-target effects of the *ranBP1-shRNA1* because the defects were fully rescued by co-electroporation with a vector encoding shRNA-resistant human *ranBP1* (Figure 5A). These findings indicate that inhibition of RanBP1 induces a significant delay in radial neural migration.

To investigate whether the high number of electroporated cells present in the VZ and SVZ was due to abnormal neuronal precursor cell (NPC) proliferation, we studied the expression of Ki67, a proliferation marker that is upregulated in all active phases of the cell cycle. At E16.5, the number of cells expressing Ki67 was similar in brains electroporated with *ranBP1-shRNA1* and shCTL (Figures 5C and 5D). To test whether the neural migration phenotype observed in *ranBP1* knockdown cortices was due to a delay of NPCs in exiting the cell cycle, E15.5 pregnant mice electroporated either with *ranBP1-shRNA1* or shCTL were injected with 5-ethynyl-2′-deoxyuridine (EdU) to label proliferating NPCs. After 24 hr, embryos were collected and brain sections were co-stained for EdU and Ki67. No differences were observed in the number of EdU^+^, Ki67^−^ cells between *ranBP1-shRNA1* and shCTL embryos, indicating that inhibition of RanBP1 expression does not affect the exit of NPCs from the cell cycle (Figures 5E and 5F). Thus, the accumulation of neurons in the VZ and SVZ and the abnormal radial migration observed in cortices electroporated with *ranBP1*-shRNA1 was not due to NPC proliferation defects.

## Discussion

Nucleocytoplasmic transport of proteins and RNAs influences a broad range of signaling and transduction pathways in eukaryotes. Our work reveals that in neurons the nucleocytoplasmic transport pathway regulates polarity because it is required for both radial migration *in vivo* and axonogenesis in cultured neurons. In cultured neurons, we find that nucleocytoplasmic transport and, in particular, RanBP1 regulate the cytosolic level of the key polarity cargo, LKB1, which promotes axon specification through microtubule regulation (Barnes and Polleux, 2009) and Golgi regulation downstream of STK25-GM130 (Matsuki et al., 2010). Thus, our results indicate that regulated export and unloading of LKB1 from the nucleocytoplasmic transport machinery is a key regulatory step of axonogenesis. Furthermore, we find that the requirement for the Ran pathway during axonogenesis is conserved through evolution.

### The Nucleocytoplasmic Transport Machinery Regulates Neuron Polarity

RanBP1 terminates nuclear export by promoting cargo release from the export complex and at the same time catalyzing the conversion of RanGTP into RanGDP. In cultured cortical neurons, the localization of the nucleus is strongly asymmetric, such that most of the cytosol, including organelles, is found proximal to the nascent axon. This suggests that most RanBP1-dependent cargo unloading occurs next to the nascent axon. Depending on the coefficient of diffusion of the export complexes and that of the cargoes exported to the cytosol, it is possible that in cortical neurons, the nucleocytoplasmic pathway initiates polarity by promoting the local accumulation of cargoes, including that of the polarity factor LKB1. Our finding that RanBP1 overexpression increases the amount of LKB1 detected in this region supports this hypothesis. In addition to its cytosolic localization, a fraction of RanBP1 is associated with the TGN. However, our attempts to identify the domain in RanBP1 that mediates this association were unsuccessful. Although it is possible that RanBP1 is modified in the Golgi, whether this event influences nucleocytoplasmic transport remains unclear.

Ran can be detected at the tip of developing neurites (Chen et al., 2017), and previous work performed in vertebrate sciatic nerve axons has shown that RanBP1 functions as part of a retrograde transport that signals to the nucleus to regulate the response to injury (Yudin et al., 2008). Consistent with Ran regulation taking place during neurite outgrowth and axonogenesis, we detected RanBP1 in the cytosol, neurites, and the nascent axon in cultured cortical neurons. It is therefore possible that during axonogenesis, RanBP1 functions as part of a retrograde pathway that involves LKB1. It will be interesting to test whether LKB1 is one of the cargoes that is regulated by the importin-β/α-dynein complex in response to nerve injury in peripheral neurons.

We also note that LKB1 is expressed in the primary cilium (Mick et al., 2015), raising the possibility that LKB1 is among the cargoes that mediate RanGTP/RanBP1 function during cilium initiation (Fan et al., 2011).

Next to axonogenesis, we find that RanBP1, and therefore the nucleocytoplasmic transport machinery, is required for radial migration during cortex development. LKB1 and STK25/GM130 have been shown to regulate radial migration in the developing cortex (Asada et al., 2007, Matsuki et al., 2013), and it is therefore possible that RanBP1-dependent export of this kinase mediates part of the RanBP1 radial migration phenotype. It is also possible that the availability of cargoes other than LKB1 is regulated by RanBP1 during this early step of neuron polarization.

### RanBP1 Regulates Neuron Polarity through Golgi Regulations

Our work demonstrates that during the establishment of neuronal polarity, a functional cross-talk takes place between two major trafficking pathways: the nucleocytoplasmic transport pathway and trafficking associated with the Golgi apparatus. The Golgi apparatus is an important regulator of neuron morphogenesis. It is located on the basal side of the migrating neuron progenitors, when post-mitotic neurons acquire their polarity (de Anda et al., 2005, Distel et al., 2010, Umeshima et al., 2007). The Golgi then relocates to the side of the apical leading edge, where it invades the apical dendrite (de Anda et al., 2010, Zhao et al., 2015). Interfering with the localization of the Golgi during this phase compromises cortex development (Horton et al., 2005), and Golgi condensation downstream of STK25 correlates with ectopic axonogenesis (Matsuki et al., 2010). However, at present it is not clear exactly how Golgi condensation may influence axonogenesis. Nevertheless, our finding that the loss of axon and the induction of hyperbranched neurites observed in RanBP1-deficient cortical neurons can be rescued by expressing STK25 indicates that most of RanBP1 function during axonogenesis and neurite branching is mediated through LKB1. It will be interesting to test whether a function for the nucleocytoplasmic transport pathway during polarity is limited to neurons or whether it extends to other cell types.

## STAR★Methods

### Key Resources Table

**Table:** 

REAGENT or RESOURCE	SOURCE	IDENTIFIER
**Antibodies**		

Rabbit polyclonal anti-Ki67	Abcam	ab15580; RRID:AB_443209
Chicken polyclonal anti-GFP	Abcam	ab13970; RRID:AB_300798
Rabbit polyclonal anti-RanBP1	Sigma-Aldrich	SAB1411184
Rabbit polyclonal anti-RanBP1	Cell Signaling	8780
Rabbit polyclonal anti-RanBP1	Abcam	ab97659; RRID:AB_10680142
Chicken polyclonal anti-Tau	Abcam	ab75714; RRID:AB_1310734
Mouse monoclonal anti-Tau	Millipore	mab3420; RRID:AB_94855
Mouse anti-TGN38	NovusBio	NB300-575SS
Mouse anti-MAP2	Abcam	ab11267; RRID:AB_297885
Rabbit anti-MAP2	Millipore	ab5622; RRID:AB_91939
Mouse anti-LKB1	NovusBio	NBP2-14835SS
Rabbit anti-Ran	Sigma-Aldrich	SAB4502579; RRID:AB_10747705
Mouse anti-RanGAP1	Santa Cruz	sc-28322; RRID:AB_2176987
Mouse anti-Myc	Santa Cruz	9E10
Chicken anti-Tuj1	Abcam	ab107216
mouse anti-Cathepsin D	BD Transduction Lab	610800; RRID:AB_CVCL_G671
mouse anti-mitofusin	Abcam	ab126575
mouse anti-BIP	BD Transduction Lab	610978; RRID:AB_11141234
rabbit anti-Transferrin Receptor	Abcam	ab84036; RRID:AB_10673794

**Bacterial Strains- Plasmids**		

*shRNA1-3 RanBP1*	pSuper GFP plasmid	Oligoengine
*shCTL shRNA*	Egan et al., 2013	pSuper GFP plasmid
Human RanBP1	pCAG-IRES-RFP	This paper
Kruppel-Gal4	This paper	N/A
RanBP1::MYC_YQRL	This paper	CMV-GFP plasmid
humanRanB1::GFP_YQRL	This paper	CMV-GFP plasmid
human RanB1(E37K)::GFP_YQRL	This paper	CMV-GFP plasmid
Mouse LKB1 cDNA	Barnes et al., 2007	pCAGGS
Mouse STRAD-α	Barnes et al., 2007	pCAGGS
Mouse STK25	Matsuki et al., 2010	pCAGGS-RFP
RanBP1-Myc-DDK	Origene	RR201578
RanBP1-GFP	Origene	RG223305

**Critical Commercial Assays**		

Rat Neurons Nucleofector kit	Lonza	VSPI-1003
EndoFree plasmid purification kit	QIAGEN	12362
Lipofectamine RNAiMAX reagent	Thermo Fisher	13778030
SYBR Mesa Blue Kit	Eurogentec	N/A

**Experimental Models: Cell Lines**		

PC12 cells	ATCC	CRL-1721
Primary rat cortical neurons	This work	N/A
Primary mouse cortical neurons	This work	N/A

**Experimental Models: Organisms/Strains**		

Mouse pups, E13.5	Charles River	C57BL/6
Drosophila RNAi lines	Vienna Drosophila RNAi Centre	N/A
Rat pups, P0	UCL facility	N/A

**Oligonucleotides**		

siRNAs RAN (rat)	Sigma	NM_053439
siRNAs RanBP1 (rat)	Sigma	NM_001108324
siRNA RCC1(rat)	Sigma	NM_001128189
siRNA LKB1(rat)	Sigma	NM_001108069
siRNA cntrl (rat)	Sigma	SIC001
*RAT GAPDH*	Eurofins Genomics	N/A
For: 5′-GACATGCCGCCTGGAGAAAC-3′		
Rev: 5′-AGCCCAGGATGCCCTTTAGT-3′		
RAT RAN	Eurofins Genomics	N/A
For: 5′-AGGACCCATCAAGTTCAACG-3′		
Rev: 5′TTCACACACGCGTACCAGAT-3′		
RAT RanBP1:	Eurofins Genomics	N/A
5′-GTGCCAACCACTACATCACG-3′		
5′-TGCCTGATCCTGCTTTCTTT-3′		
*RAT Rcc1*	Eurofins Genomics	N/A
5′-CCGGGAAAGTGGAACTACAA-3′		
5′-ACCTTTACCACCTGCGTGTC-3′		
RAT LKB1	Eurofins Genomics	N/A
5′-CCTGCAGAGAAAACCCAGAG-3′		
5′-GCAGCTTCAAGTTTCCCAAG-3′		

**Software and Algorithms**		

ImageJ	NIH GOV	N/A
LAS AF software	Leica	N/A

**Other**		

Sequencing	Eurofins Genomics	N/A

### Contact for Reagents and Resource Sharing

Further information and requests for resources and reagents should be directed to and will be fulfilled by the Lead Contact, Franck Pichaud (f.pichaud@ucl.ac.uk).

#### Drosophila

For the F_1_ recessive RNAi screen in the Bolwig’s organ, RNAi lines [Vienna *Drosophila* RNAi Centre (VDRC)] (Dietzl et al., 2007), were expressed using a Kruppel-Gal4 driver (a generous gift from Fernando Casares, Sevilla, Spain). The Bolwig’s nerve was scored using the artificial neuronal 3 × P3-GFP transgene (Berghammer et al., 1999).

#### Mice

All experiments were conducted with University College London ethical committee approval. E13.5 timed-pregnant mice were handled and electroporated as previously described in (Nitarska et al., 2016). Pregnant mice were sacrificed 72h following surgery.

#### Primary cultures of rat cortical neurons

All experiments were conducted with University College London ethical committee approval. Cells were isolated form P0 cortices and electroporated before plating using the Nucleofector kit (Lonza). Briefly, 1 × 10^6^ cells were resuspended in 100 μL of Nucleofector solution containing 3 μg of an expression vector or a pool of three gene-specific siRNAs (10 nM of each siRNA, 30 nM total), or a combination of expression vectors plus siRNAs. siRNAs targeting *ran* (NM_053439), *ranBP1* (NM_001108324), *rcc1* (NM_001128189) and *LKB1* (NM_001108069), and a siRNA negative control (MISSION® siRNA Universal Negative Control, SIC001) (Sigma).

### Method Details

#### Immunological Methods

Cells were washed (PBS), fixed (PBS, 3% paraformaldehyde; 15 min), washed (PBS), permeabilized in PBS-T (PBS plus 0.3% Triton X-100; 20 min), blocked (PBS-T plus 5% goat serum; 15 min) and incubated with primary antibodies overnight at 4°C and appropriate secondaries antibodies (Jackson ImmunoResearch, dilution 1:200; 1 h, room temperature). The primary antibodies used were: rabbit anti-RanBP1 (1:500, SAB1411184, Sigma-Aldrich), rabbit polyclonal anti-RanBP1 (#8780, Cell Signaling), rabbit polyclonal anti-RanBP1 (1:4000, ab97659 Abcam), chicken anti-Tau (1:1000, ab75714 Abcam), mouse anti-Tau (1:1000, mab3420, Millipore), mouse anti-TGN38 (1:100, NB300-575SS, Novusbio), mouse anti-MAP2 (1:1000, ab11267, Abcam), rabbit anti-MAP2 (1:1000, ab5622 Millipore), mouse anti-LKB1 (1:100, NBP2-14835SS, Novusbio), rabbit anti-Ran (GTP+GDP) (1:1000, SAB4502579, Sigma-Aldrich), mouse anti-RanGAP1 (1:200, sc-28322, Santa Cruz Biotechnology), mouse anti-Myc (1:500, 9E10, Santa Cruz), chicken anti-GFP (1:1000, ab13970, Abcam) and chicken anti-Tuj1 (1:1000, ab107216, Abcam).

#### Tissue preparation and Immunostaining

Embryonic brains were processed as previously described in (Nitarska et al., 2016). The following primary antibodies were used: rabbit anti-Ki67 (Abcam ab15580), chicken anti-GFP (Abcam ab13970), rabbit anti-RanBP1 (1:500, SAB1411184, Sigma-Aldrich).

#### Radial neural migration analysis

Radial migration analysis of embryos electroporated in utero with the indicated GFP vectors was performed as described previously (Hand et al., 2005) using ImageJ and excel macro.

#### Cell cycle index analysis

Cell cycle exit index was determined using EdU/Ki67 immunolabelling. For EdU incorporation E15.5 timed pregnant females received an intraperitoneal injection of EdU (Invitrogen) at a dose of 20 mg/kg body weight. After 24h, coronal sections were stained for EdU incorporation using the Click-iT EdU cocktail and immunolabelled with the Ki67 antibody. All imaging and image analysis were performed blind. Quantification of labeled cells within the cortical wall was performed by dividing the images into bins 100 μm wide. The bins spanned the entire coronal section from the ventricular surface to the pia. EdU^+^/Ki67^-^ cells and total number of EdU^+^ cells were counted per each bin. Cell cycle exit index was calculated as the percentage of EdU^+^/Ki67^-^ cells over the total number of EdU^+^ cells.

#### Molecular Biology

Rat *RanBP1-Myc-DDK* (RR201578) and human *RanBP1-GFP* (RG223305) expression plasmids were purchased from Origene. A genomic rescue transgene was generated that consists of a 1kb *ranBP1*promoter cloned upstream of the RanBP1::GFP fusion protein. Rat *RanBP1-Myc-DDK-YQRL*, and human *RanBP1-GFP-YQRL* were generated by directly ligating annealed oligos into the vector backbones using the PmeI restriction site. The *RanBP1(E37K)-GFP-YQRL* mutant was generated by site-directed mutagenesis (Quick Change Mutagenesis Kit, ThermoFisher). Mouse Par4/LKB1 and mouse STRADα plasmids were a kind gift of Franck Polleux, Columbia University, USA. For in utero electroporation experiments the oligo hairpins were ligated into pSUPER GFP plasmid (Oligoengine) using BglII/HindIII restriction sites. The shRNA sequences targeting mouse RanBP1 transcripts were as follows: *shRNA1* 5′-CGTGCAAAGCTGTTCCGGTTTGTTCAAGAGACAAACCGGAACAGCTTTGCAC3′, *shRNA2* 5′-CCCTACCCTTTAAGGTTTGTTTTTCAAGAGAAAACAAACC

TTAAAGGGTAGG-3′, *shRNA3* 5′-GGACCATCCGCCTTCTTATGATTCAAGA

GATCATAAGAAGGCGGATGGTCC-3′, *shCTL shRNA* sequence 5′-GCGTACGGGGAAACTTCGA-3′ was described previously (Egan et al., 2013).

Complete hRanBP1 coding sequence was subcloned into pCAG-IRES-RFP vector using NotI and EcoRI.

#### Western blot and quantitative real-time PCR

PC12 cells were electroporated and harvested 48h after seeding to be analyzed by either western blot or qRT-PCR. Cells were lysed with ice-cold extraction buffer (50 mM Tris-HCl (pH 7.4), 150 mM NaCl, 5 mM EDTA, 0.1% NP-40). Samples were centrifuged at 20,000 g for 10 min at 4°C and protein content determined by the BCA protein assay kit. The total quantity of protein (40 μg) was subjected to gel electrophoresis on a 10% SDS-PAGE, immunoblotted and probed for rabbit anti-Ran (GTP+GDP) (1:1000, SAB4502579, Sigma-Aldrich) or rabbit anti-RanBP1 (1:1000, SAB1411184, Sigma-Aldrich), together with mouse anti-GFP (1:1000, ab1218, Abcam). Membranes were incubated with the appropriate anti-mouse (1:10000, IR-680, LI-COR Biosciences) and anti-rabbit (1:10000, IR*-*800, LI-COR Biosciences) antibodies. Protein signal was detected by Odyssey infrared imaging system LI-COR Biosciences (LI-COR).

Total RNA was extracted using Trizol reagent (Invitrogen), and 1 μg of total RNA was used for cDNA synthesis using SuperScript RT II (Invitrogen). Real-time qPCR was performed using SYBR Mesa Blue Kit (Eurogentec). The following primers were used: *GAPDH* 5′-GACATGCCGCCTGGAGAAAC-3′ (forward) and 5′-AGCCCAGGATGCCCTTTAGT-3′ (reverse); *Ran* 5′-AGGACCCATCAAGTTCAACG-3′ (forward); 5′TTCACACACGCGTACCAGAT-3′ (reverse); *RanBP1* 5′-GTGCCAACCACTACATCACG-3′ (forward); 5′-TGCCTGATCCTGCTTTCTTT-3′ (reverse); *Rcc1* 5′-CCGGGAAAGTGGAACTACAA-3′ (forward); 5′-ACCTTTACCACCTGCGTGTC-3′ (reverse). LKB1 5′-CCTGCAGAGAAAACCCAGAG-3′ (forward); 5′-GCAGCTTCAAGTTTCCCAAG-3′ (reverse). Statistical significance was calculated using an unpaired t test with significance at p < 0.05.

#### Biochemical Analysis

Brains obtained from rat pups (P0) were processed on ice with a Dounce homogenizer in 1 mL ice-cold homogenization buffer (HB: 250 mM sucrose; 20 mM HEPES-HCl, pH 7.4; 1 mM EDTA; 5 mM MgCl2), supplemented with 0.2% protease inhibitors (Sigma-Aldrich) and centrifuged at 900 g for 5 min at 4°C. The supernatant was recovered and the pellet re-suspended in 500 ul HB, re-homogenized and centrifuged as described above. The supernatants were pooled to yield a post-nuclear supernatant (PNS). PNSs were fractionated by sequential centrifugation through two sucrose gradients, essentially as previously described (Ferraro et al., 2007).

#### Brefeldin A treatments

Brefeldin A was obtained from Sigma. Primary rat neurons were treated with brefeldin A (1 μg/ml for 90 min) to induce Golgi dispersion. Cells were then fixed and processed for immunofluorescence.

### Quantification and Statistical Analysis

#### Axon quantification

The mean percentages of 4 stage neurons with 0, 1 or multiple axons were determined for each condition. After converting percentages to arcsin values, two-tailed, unpaired, t tests were performed comparing knockdown/mutants to control.

#### Morphological characteristics of neurons

Sholl analysis was used to characterize the morphology of imaged neurons. Statistics were calculated by unpaired, two-tailed Student’s t test.

#### Western blot quantification

Densitometry analysis of signal intensity in western blotting was performed with a Student’s t test.

#### Neuronal migration

Neuronal migration was analyzed using two way Anova with Tukey’s post test.

Neuronal progenitor proliferation index was measured as GFP^+^Ki67^+^ / GFP^+^ cells and the rate of cell cycle exit was measured as the ratio of Edu^+^Ki67^-^ / Edu^+^ cells.

#### IF Colocalization

to analyze the colocalization of the proteins of interest and cellular compartments, the Pearson’s correlation coefficients were used. The Pearson correlation is +1 in the case of a perfect direct (increasing) linear relationship (correlation), and −1 in the case of a perfect decreasing (inverse) linear relationship (anticorrelation). The Person correlation values were calculated by the pixel intensity- spatial correlation analysis method using confocal images and ImageJ processing program.

#### Real Time PCR

Statistical significance was calculated by comparing the normalized expression ratios to 1 and using one sample Student’s t test.

All analyses were performed using data from at least three biological replicates. In all cases, a p value of ≤ 0.05 was considered to be significant.

## Supplemental Information

Document S1. Figures S1–S7

Table S1. Candidate Genes Identified in the Bolwig’s Organ, Related to Figure 1

Document S2. Article plus Supplemental Information
