# Supplementary material for: RanBP1 Couples Nuclear Export and Golgi Regulation through LKB1 to Promote Cortical Neuron Polarity
Source: Cell Rep. 2018 Sep 4;24(10):2529–2539.e4. doi: 10.1016/j.celrep.2018.07.107 (PMC6137820; doi:10.1016/j.celrep.2018.07.107)
Supplement: Document S1. Figures S1–S7 [file mmc1.pdf]

Cell Reports, Volume *24*

Supplemental Information

RanBP1 Couples Nuclear Export and Golgi Regulation through LKB1 to Promote Cortical Neuron Polarity

Chiara Mencarelli, Justyna Nitarska, Tim Kroecher, Francesco Ferraro, Katherine Massey, Antonella Riccio, and Franck Pichaud

Figure S1. The Ran-pathway is required for nerve growth in *Drosophila*

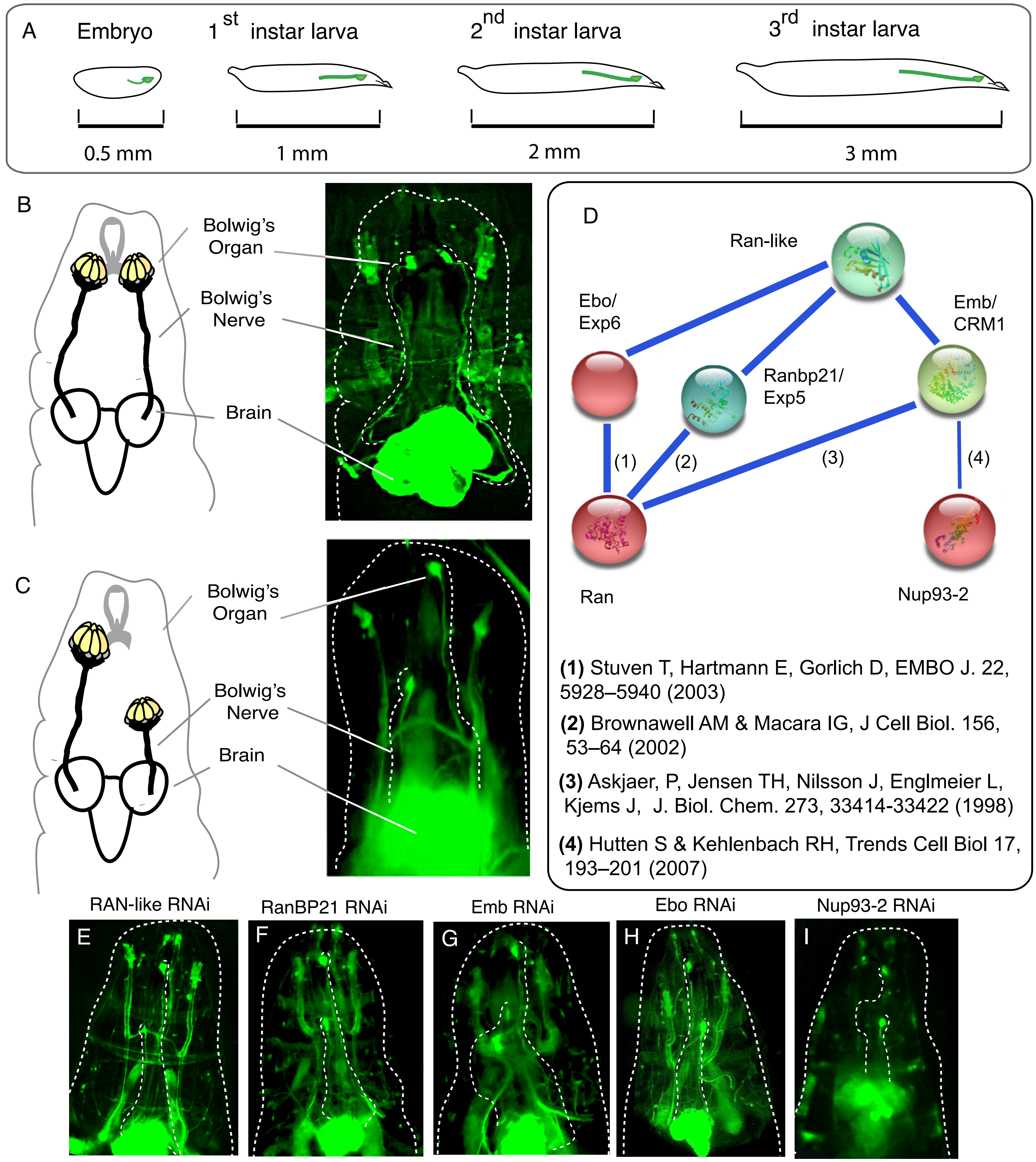

Figure S1: The Ran-pathway is required for axon growth in *Drosophila* - Related to Figure 1

(A) *Drosophila* larval development from the embryo to the 3rd instar larval stage. The Bolwig's organ (BO) is shown in green. The Bolwig's nerve (BN) connects the BO to the optic lobe early during embryogenesis and elongates as the larva grows. (B-C) Images of 3rd instar larvae, showing wild-type BN (B) and the short BN phenotype associated with *ran* RNAi mediated knockdown (C). Ran-related gene network identified in the BN screen. The lines between the circles indicate experimental evidence of an association/interaction, as generated by the STRING server (*string-db.org*). (E-I) Images of larvae of the BN showing phenotypes associated with the knockdown of *ran-like* (E), *ranBP21*, *emb* (*CRM1*), *ebo (Exp-6)* (H), *nup-93-2* (I). In all cases, the phenotype is a shorter BN indicating defects in axon growth.

Figure S2: A fraction of RanBP1 is assocaited with the Golgi

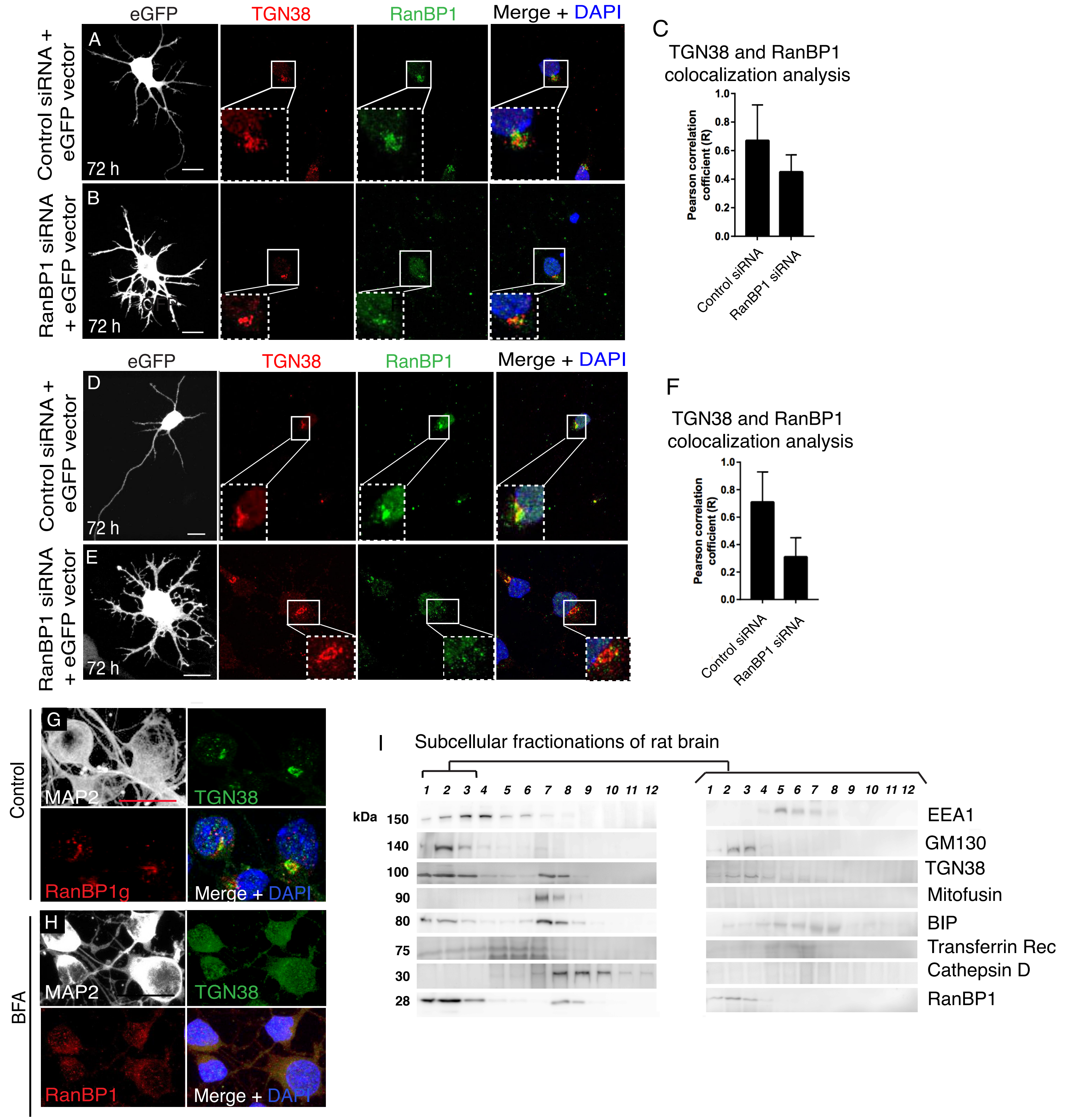

Figure S2: A fraction of RanBP1 is associated with the Golgi - Related to Figure 1

(A) 72h rat cortical neuron transfected with control siRNAs and the *eGFP*-reporter. (B) 72h neuron transfected with siRNAs *RanBP1* and the *eGFP*-reporter plasmid. TGN38 labels the Golgi, RanBP1 (Cell signaling). (C) Quantification of the RanBP1 staining associated with the TGN. Pearson's correlation coefficients of 0.67 ± 0.25 (control) and 0.45 ± 0.12 (RanBP1 siRNA) (D) 72h neuron transfected with control siRNAs and the *eGFP*-reporter. (E) 72h neuron transfected with siRNAs *RanBP1* and the *eGFP*-reporter plasmid. TGN38 labels the Golgi, RanBP1 (Sigma). Scale bar, 10 µm. (F) Quantification of the RanBP1 staining associated with the TGN. Pearson's correlation coefficients of 0.71 ± 0.22 (control), 0.31 ± 0.14 (RanBP1 siRNA) (n = at least 8 neurons for each condition). (G-H) Distribution of TGN38 and RanBP1 in wild-type and Brefeldin A-treated (H) in 72 h cortical neurons. Scale bar, 10 µm. (I) Immunoblot analyses of consecutive cell fractionation from by rat brain showing that a fraction of RanBP1 co-sediments with Golgi markers (GM130 and TGN38). EEA1 is an early endosome marker, GM130 and TGN38 are Golgi markers; Mitofusin is a mitochondria marker; BIP is an endoplasmic reticulum marker; Transferrin receptor (TfR) is an early recycling endosome marker; Cathepsin D is a lysosomal marker.

Figure S3: Validation of the siRNA targeting Ran, Rcc1, RanBP1 and LKB1

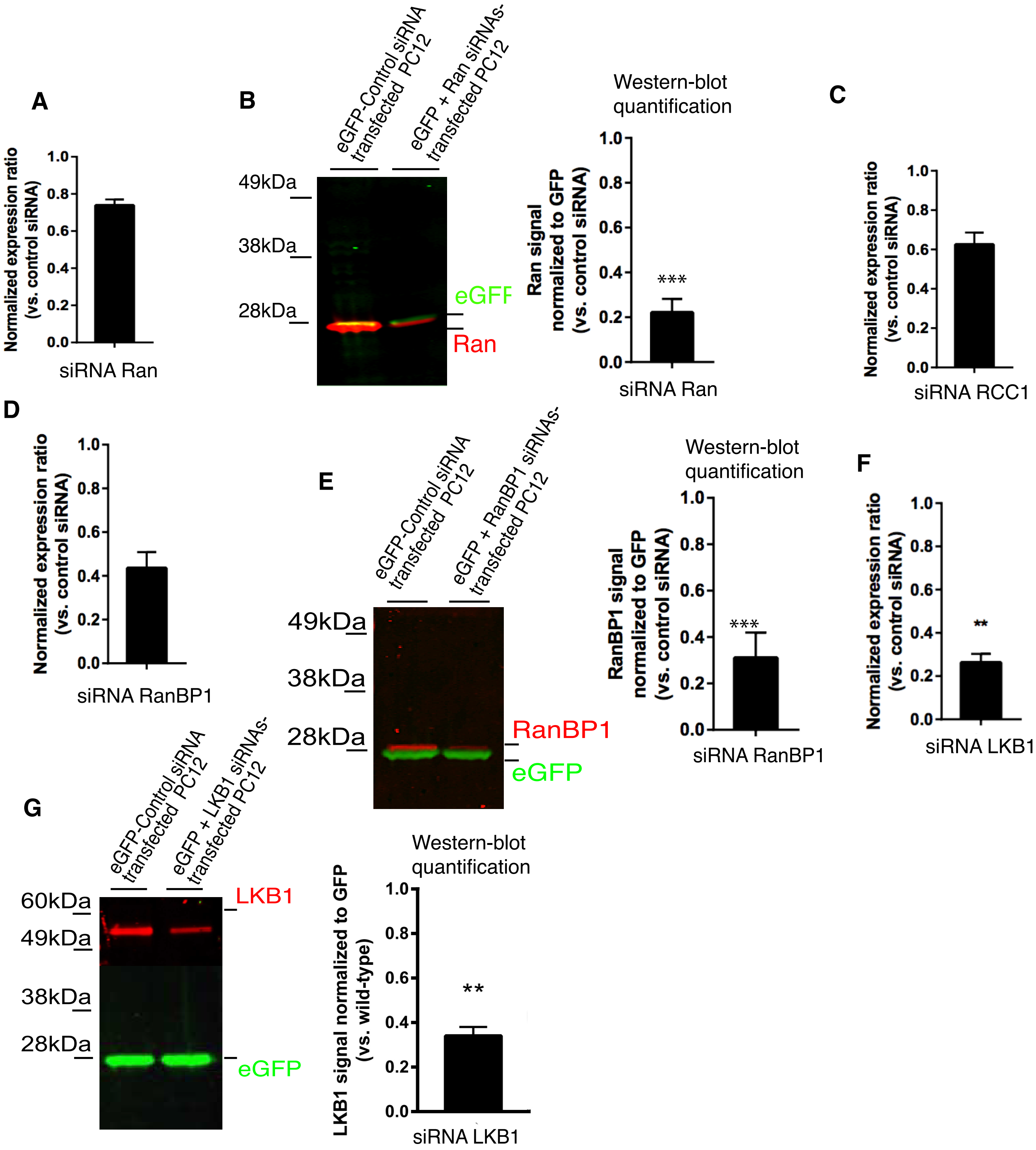

Figure S3: Validation of the siRNA targeting Ran, Rcc1, RanBP1 and LKB1- Related to Figure 2

(A) Real-time PCR bar graph for *ran* siRNAs transfected PC12 (n= 3 independent mRNA extracts). Normalized expression ratio (mean +/- SEM) compared to GAPDH: *siRNA ran*: 0.739±0.031 (p-value <0.01). Error bar represents SEM. (B) Western-blot and gel densitometry showing Ran protein levels in *ran* siRNA transfected PC12 cells compared to control cells. Western-blot quantification: *siRNA ran* = 0.221+/-0.061, p-value < 0.001. (C) Real-time PCR bar graph for *rcc1* siRNAs transfected PC12. Normalized expression ratio (mean +/- SEM) compared to GAPDH*: siRNA rcc1* = 0.625 +/- 0.061, p-value < 0.01. Error bar represents SEM. (D) Real-time PCR bar graph for *ranBP1* siRNAs transfected PC12 cells. Normalized expression ratio (mean +/- SEM) compared to GAPDH: *siRNA RanBP1* = 0.435 +/- 0.074 (p-value <0.001). Error bar represents SEM. (E) Western blot and gel densitometry of *ranBP1* mRNA levels in *ranBP1* siRNAs transfected PC12 cells when compared to control (n= 3independent mRNA extracts). Western-blot quantification: *siRNA ranbp1* = 0.31+/-0.11, p-value < 0.001. (F) Real-time PCR bar graph for *LKB1* siRNAs transfected PC12 cells. Normalized expression ratio (mean +/- SEM) compared to GAPDH: *siRNA LKB1* = 0.26 +/- 0.039 (p-value <0.001). Error bar represents SEM. (G) Western blot and gel densitometry of *lkb1* mRNA in *lkb1* siRNAs transfected PC12 cells (n= 3 independent mRNA extracts). Western-blot quantification: *siRNA lkb1* = 0.34+/-0.01, p-value < 0.001.

Figure S4: RanBP1 deficient neurons fail to form the initial segment

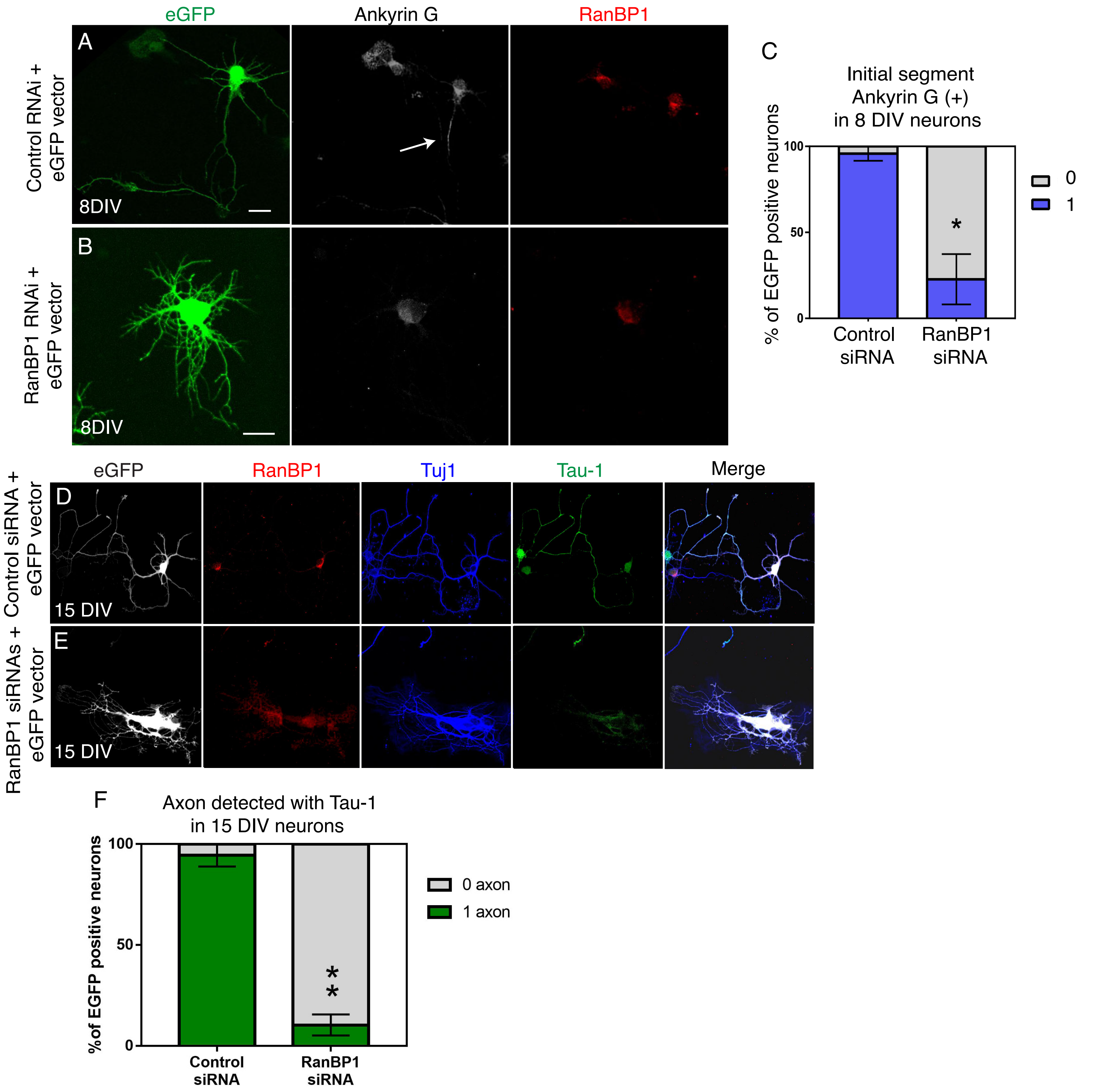

Figure S4: RanBP1 deficient neurons fail to form the initial segment - Related to Figure 2

(A) Representative *DIV 8* rat cortical neuron transfected with scrambled siRNAs and the *eGFP*-reporter and (B) transfected with siRNAs targeting *RanBP1* together with the *eGFP*-reporter plasmid, and stained for AnkyrinG. (C) The mean percentage of neurons with 0 or 1 AIS over three independent replicates was determined for each condition (Control siRNA, 95.83 ± 4.17% with 1 AIS; RanBP1siRNA, 22.73 ± 14.61% with 1 AIS). After converting percentages to arcsin values, two-tailed, unpaired, t-tests were performed (p value = 0.0085). (D) Representative image of a cortical neuron co-transfected with scrambled siRNA and the eGFP-reporter plasmid and (E) transfected with rat-specific siRNAs targeting *ranBP1*, and the eGFP-reporter plasmid at 15 days after electroporation. Transfection with siRNAs was repeated at 5 and 10 days after the initial electroporation to ensure effective knockdown over time. (F) The mean percentage of neurons with 0 or 1 axon over three independent replicates was determined for each condition: control siRNA at 15 DIV (n=22 neurons), RanBP1 siRNA at 15 DIV (n=20 neurons). After converting percentages to arcsin values, a two-tailed, unpaired, t-test was performed, comparing control siRNA to RanBP1 siRNA (p=0.000396).

Figure S5: RanBP1 function during axogenesis depends on Ran

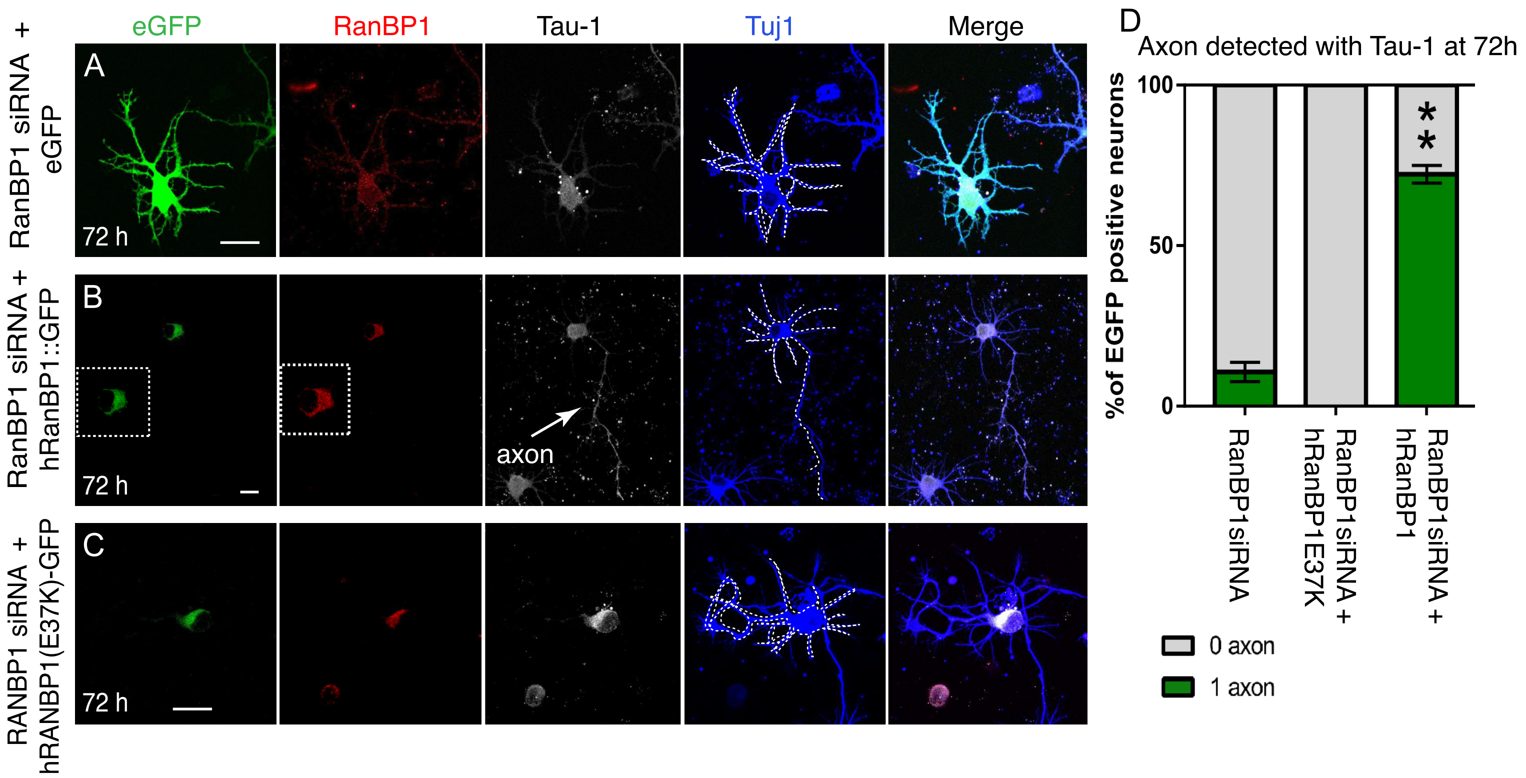

Figure S5: RanBP1 function during axogenesis dependents on its binding to Ran - Related to Figure 2

(A) 72 h neuron deficient for *ranBP1* and (B) 72h cortical neuron deficient for *ranBP1* expressing the rescue construct hRanBP1::GFP. The inserts in (B) show higher magnifications of hRanBP1::GFP localization and RanBP1 immunostaining. The hRanBP1::GFP fusion protein rescues neuronal polarity in 72% of transfected neurons (Tau) (*n* = 36, 72 h neurons from three independent experiments). The white arrow in (B) points to the rescued axon. (C) Re-introducing a mutated form of ranBP1 (hRanBP1-E37K-GFP) in RanBP1 siRNA knockdown neurons does not rescue neuronal polarity (*n* = 22, 72 h neurons from three independent experiments). (D) The mean percentages of stage 4 neurons with 0 or 1 axon over three independent replicates were determined for each condition: RanBP1 siRNA (n = 38 neurons), RanBP1 siRNA rescued with hRanBP1-GFP (n = 36 neurons) and hRanBP1-E37K-GFP (n = 22 neurons). After converting percentages to arcsin values, two-tailed, unpaired, t-tests were performed, comparing RanBP1 siRNA to RanBP1 siRNA rescued with hRanBP1-GFP (p = 0.0002) or hRanBP1-E37K-GFP (p = 0.326).

Figure S6: Localization of RanBP1, Ran and RanGAP in the developing cortex

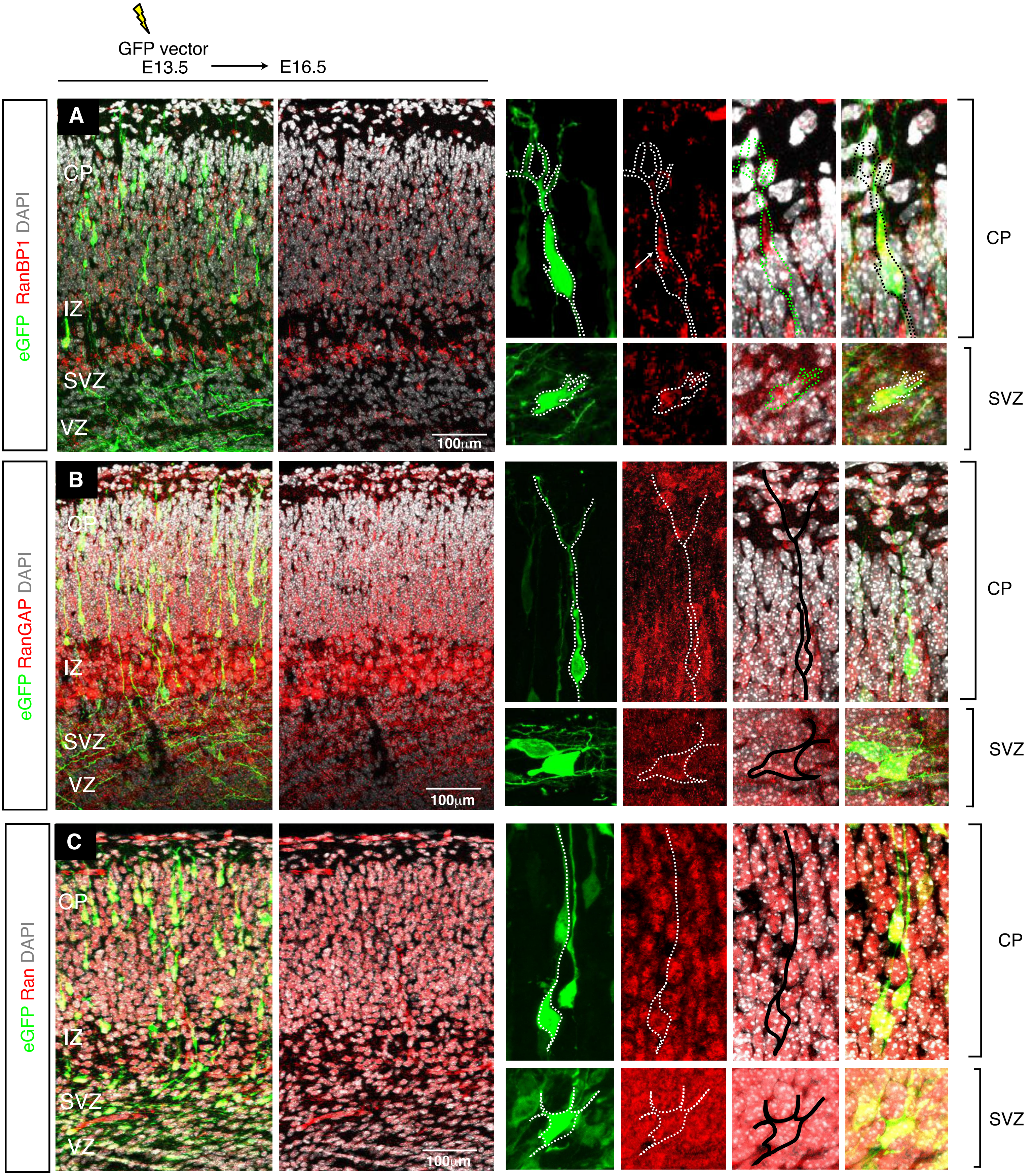

Figure S6: Localization of RanBP1, Ran and RanGAP in the developing cortex - Related to Figure 5

(A) E13.5 mice were *in utero* electroplated with shCTL-IRES-GFP and stained at E16.5 for RanBP1, (B) RanGAP and (C) Ran. Electroporated cells are green and outlined using a dashed line. Respective patterns of expression are shown in neurons in either the CP or SVZ. CP, cortical plate; IZ, intermediate zone; SVZ, sub ventricular zone; VZ, ventricular zone.

Figure S7: RanBP1 shRNA validation

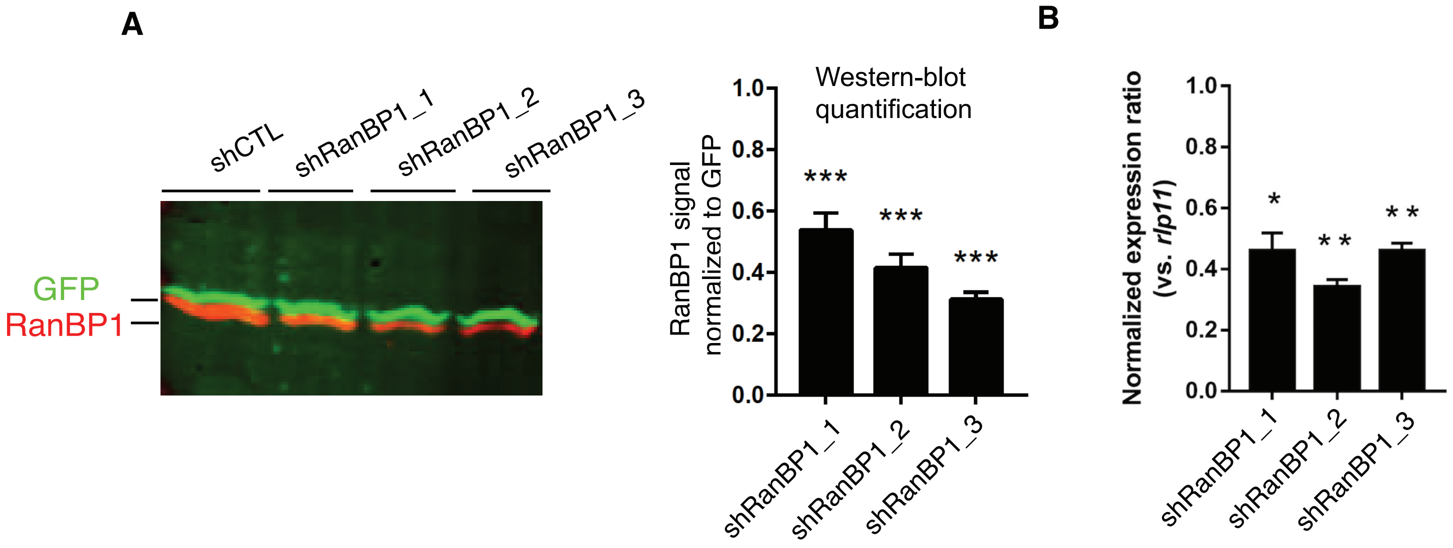

Figure S7: RanBP1 shRNA validation - Related to Figure 5

(A) Western blotting and gel densitometry to test the suitability of three RanBP1shRNA constructs for subsequent use in *in utero* electroporation experiments. NIH 3T3 fibroblasts were transfected with RanBP1shRNA constructs and collected for Western blotting. SDS-PAGE gels were stained for GFP and RanBP1. RanBP1 expression (normalized to GFP) was significantly reduced by each of the shRNA constructs vs. the shCTL (relative densities and p-values compared to the shCTL: shRanBP1_1, 0.55 ± 0.06 [$p < 0.01$]; shRanBP1_2, 0.41 ± 0.04 [$p < 0.001$]; shRanBP1_3, 0.31 ± 0.02 [$p < 0.0001$]). (B) qRT-PCR of RanBP1_1-3 tested in NIH 3T3 fibroblasts shows the normalized expression ratio (mean +/- SEM) compared to *rlp11*. Three independent mRNA extracts of control, shRNA1, shRNA2 and shRNA3 cells. Statistical significance was calculated by comparing the normalized expression ratios to 1 using one sample t-tests (shRanBP1_1, $p = 0.0116$; shRanBP1_2, $p = 0.0016$; shRanBP1_3, $p = 0.0022$). *$p < 0.05$, **$p < 0.01$.
